# Supplementary material for: Late Holocene fast-ice dynamics around the Northern Victoria Land coast, Antarctica
Source: Nat Commun. 2026 Jan 20;17:604. doi: 10.1038/s41467-025-67781-7 (PMC12820077; doi:10.1038/s41467-025-67781-7)
Supplement: Supplementary file 1 — Supplementary Information [file 41467_2025_67781_MOESM1_ESM.pdf]

Late Holocene fast-ice dynamics around the Northern Victoria Land coast, Antarctica

T. Tesi[1]., M.E. Weber[2], F. Muschitiello[3,4], D. Dutta[3,5], S.T. Belt[6], C. Pambianco[1,7], A. Di Roberto[8], L. Silva[9], K. Gariboldi[9], C. Morigi[8], F. Battaglia[1], E. Colizza[10], L. De Santis[11], A. Gallerani[12], G. Aulicino[13,1], L. Langone[1], P. Giordano[1]

(1) Istituto di Scienze Polari - Consiglio Nazionale delle Ricerche ISP-CNR, Via P. Gobetti 101, 40129 Bologna, Italy
(2) Institute for Geosciences, Department of Geochemistry and Petrology, University of Bonn, 53115 Bonn, Germany
(3) Department of Geography, University of Cambridge, Cambridge, CB2 3EN, UK
(4) Centre for Climate Repair at Cambridge, Department of Applied Mathematics and Theoretical Physics, University of Cambridge, Cambridge, UK
(5) Research School of Earth Sciences, Australian National University, Canberra, ACT, Australia
(6) Biogeochemistry Research Centre, School of Geography, Earth and Environmental Sciences, University of Plymouth, Drake Circus, Plymouth, Devon PL4 8AA, UK
(7) Campus Madonna delle Piane, Università degli Studi "G. d'Annunzio" Chieti-Pescara, Via dei Vestini 31, 66013 Chieti (Chieti Scalo), Italy.

(8) Istituto Nazionale di Geofisica e Vulcanologia (INGV), Sezione di Pisa, Via Cesare Battisti 53, 56125, Pisa, Italy
(9) Department of Earth Sciences, University of Pisa, Via Santa Maria 53, 56126 Pisa, Italy
(10) Dipartimento di Matematica, Informatica e Geoscienze, Università di Trieste, Via E. Weiss 2, 34127, Trieste, Italy
(11) Istituto Nazionale di Oceanografia e di Geofisica Sperimentale, OGS, Borgo Grotta Gigante 42/c, 34010 Sgonico (Trieste), Italy
(12) Istituto di Scienze Marine, Consiglio Nazionale delle Ricerche ISMAR-CNR, Via P. Gobetti 101, 40129 Bologna, Italy
(13) Dipartimento di Scienze e Tecnologie, Università degli Studi di Napoli "Parthenope", Napoli, 80143, Italy

1. Geophysics

Echosounding sub-bottom profiles were used to locate core TR17-08 coring position, taking into account sediment geometry of Edisto Inlet[1]. Sub-bottom profiling was conducted using a Geoacoustic Geopulse 5430 with a pulse cycle of 1 and a source frequency of 3.5 kHz. The signal sampling interval is 0.83 milliseconds, corresponding to approximately 50 cm. However, the vertical resolution of these profiles is insufficient to resolve individual laminations observed in the sediment cores. Despite this limitation, acoustic reflectors with increased amplitude that thicken toward the central sector of the fjord support the interpretation that sediment core HLF17-01 represents a more expanded record compared to core TR17-08. The sub-horizontal nature of the reflectors draping the pre-existing morphology indicates that the sediment in both cores was deposited predominantly through hemipelagic settling or under conditions of very slow circulation. Low-angle unconformities bounding reflector packages may correspond to environmental changes occurring on a millennial scale[1]

2. ^{210}Pb analyses on LS23-MUC17-08

We measured ^{210}Pb in LS23-MUC17-08 (Figure S1) as described in the Methods. The record shows excess ^{210}Pb in the upper 14 cm. Derived sediment accumulation rate (SAR; Figure S1b) indicates constant sedimentation over the last century. Instead, measurements carried out on TR17-08 revealed lack of excess ^{210}Pb. This implies that the core is missing its uppermost sediments which were probably lost during the sampling of soft diatom oozes.

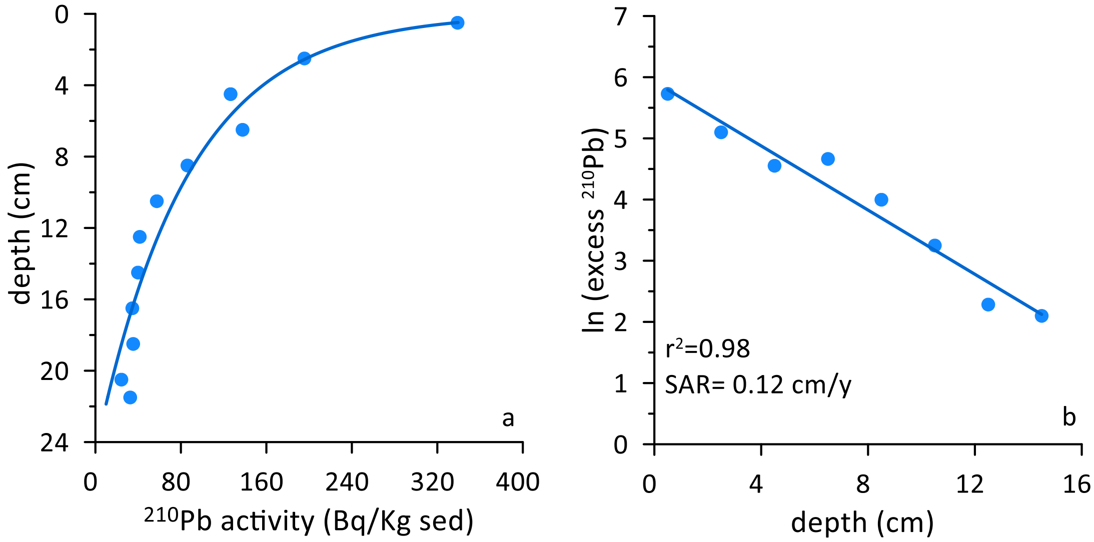

Figure S1. **^{210}Pb data of LS23-MUC17-8 record.** ^{210}Pb activity of LS23-MUC17-08 vs sediment depth (a) and linear fit of the log-transformed excess ^{210}Pb (b).

3. TR17-08 age-depth model

The age model for core TR17-08 was developed using 10 radiocarbon measurements of carbonate tests and the Mount Rittmann tephra layer[2] (Table S1) as presented in the Methods. Here we further explain how we assess the reservoir effect (ΔR). For core TR17-08, radiocarbon measurements were calibrated against the Marine20 calibration curve [3]. We leveraged the calendar age of the tephra horizon to assess the ΔR of the nearest carbonate test, located 6.5 cm below the tephra. This ΔR value was then applied to the other dates, considering that ΔR in the Ross Sea has remained relatively constant throughout the Holocene[4]. The calendar age of the Mount Rittmann tephra layer was derived from the annually resolved WAIS Divide 2014 chronology[2]. To account for the age gap, we used the sediment accumulation rate based on the ^{210}Pb analysis from core LS23-MUC17-08. The estimated ΔR of 663 ± 35 years aligns with the local reservoir correction (ΔR) of 600 ± 120 years proposed for the Ross Sea as a whole[4], adjusted to align with Marine20. However, the new ΔR has a lower uncertainty, which in turn reduces the error in the spectral power analysis.

The final age model exhibited a stable MCMC run and demonstrated consistency with the age model for core HLF17-1, as presented in Tesi et al.[5]. This alignment reinforces the reliability of the age-depth model and its application to reconstruct sedimentary histories (Figure S2).

**AWI MICADAS lab ID**	**sample ID**	**organism**	**core depth (cm)**	**^{14}C age (yBP)**	**error (y)**	**ΔR**	**modelled (cal yBP)**		
							median	from (2σ)	to (2σ)
	tephra Mount Rittmann		55.5				684	659	708
6735.1.1	TR17-08 XV 61-63	Echinoidea	62	2,036	28	663±35	700	677	734
6736.1.1	TR17-08 XIII 78-79	Echinoidea	234.5	2,312	28	663±35	1047	953	1152
6737.1.1	TR17-08 X 13-14	Ophiuroidea	463.5	2,646	24	663±35	1512	1404	1635
9346.1.1	TR17-08 X 61-63	Benthic foraminifera	512	2,601	91	663±35	1635	1525	1746
9347.1.1	TR17-08 X 66-69	Benthic foraminifera	517.5	2,943	77	663±35	1649	1540	1759
9348.1.1	TR17-08 X 74-75	Benthic foraminifera	524.5	2,967	74	663±35	1669	1558	1779
9349.1.1	TR17-08 X 75-76	Benthic foraminifera	525.5	2,977	74	663±35	1671	1561	1781
6738.1.1	TR17-08 VIII 77-78	Ophiuroidea	729.5	3,361	31	663±35	2158	2020	2285
6740.1.1	TR17-08 VI 12-13	Echinoidea	863.5	3,675	23	663±35	2448	2307	2597
6741.1.1	TR17-08 I 17-18	Scaphopoda	1369.5	4,287	23	663±35	3410	3250	3609

Table S1. Dates used to build the age-depth model of core TR17-08.

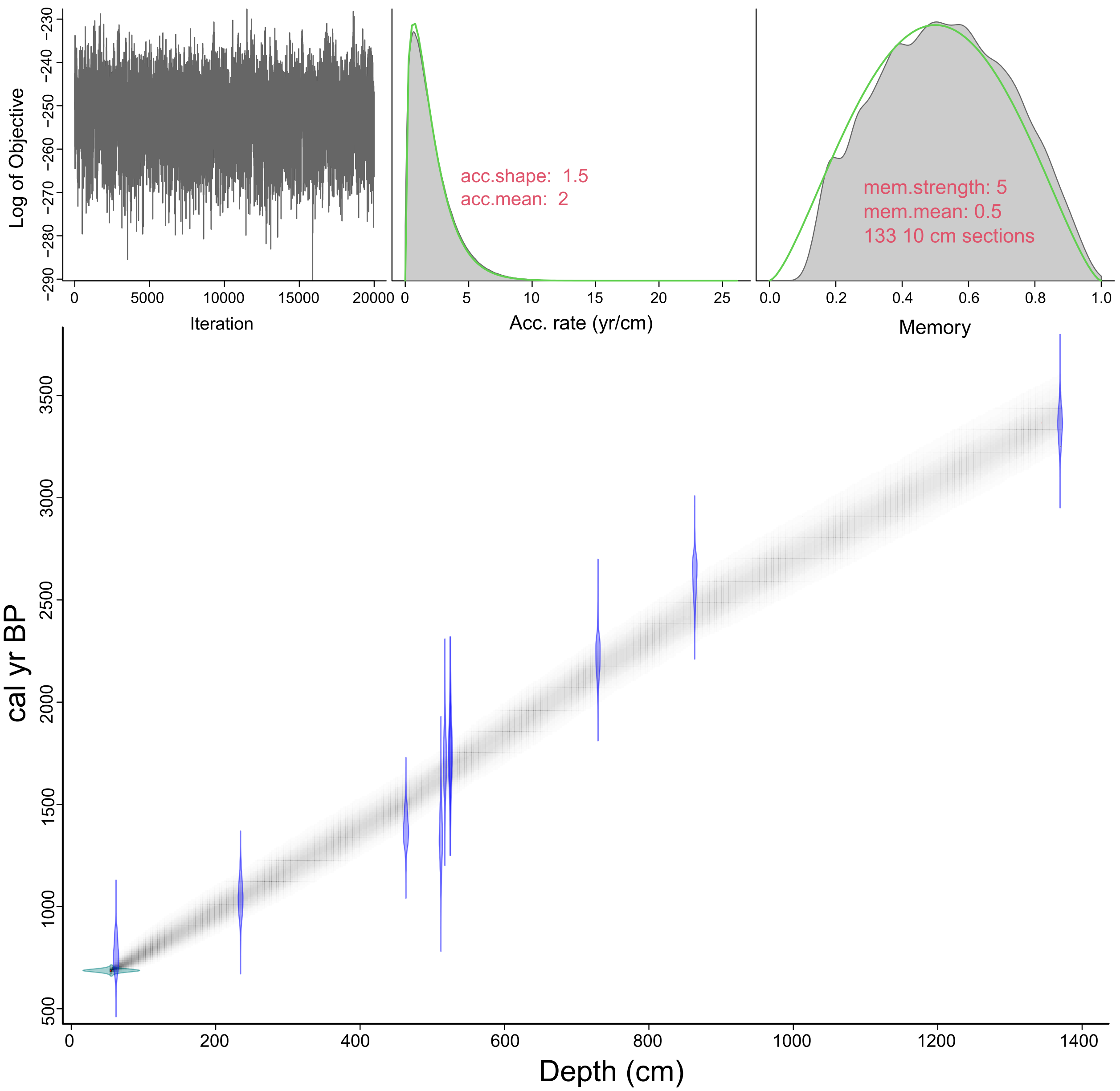

Figure S2. **Age-depth model of core TR17-08 obtained with Bacon 2.3**. Dates used in the age-model are shown in blue. Mount Rittmann tephra layer (55 cm) is displayed in green. Red line shows the median age. Shaded grey area shows the uncertainty (2σ) of the modelled ages.

3. Image analyses

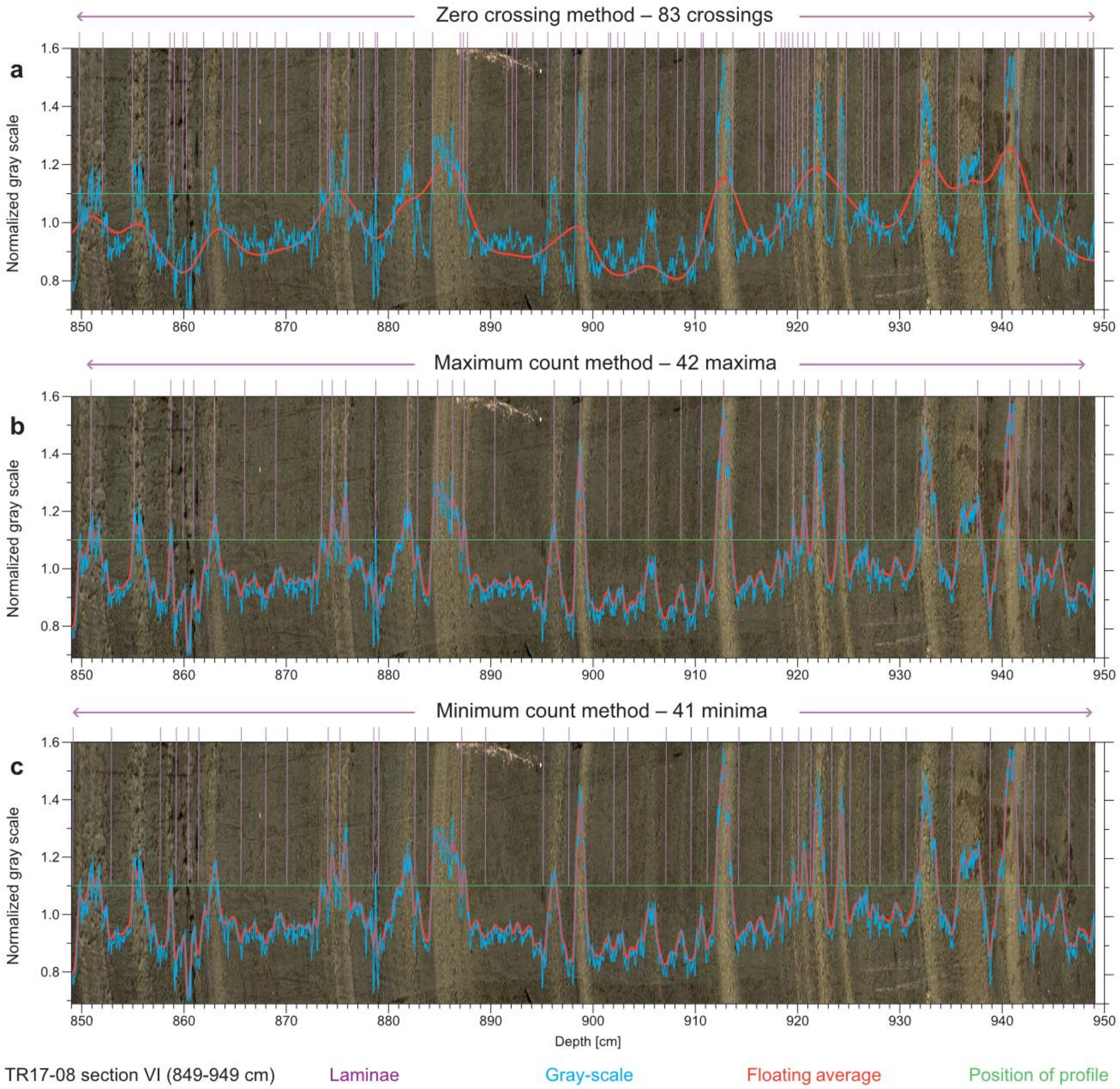

Figure S3. **Results from automated laminae recognition and counting software BMPIX/PEAK**. Example refers to core TR17-08, section VI (849-949 cm core depth) and shows three out of the five methods used. a. Zero crossing method. b Maximum count method. c Minimum count method. Top (left) and bottom (right) of the image are used to convert the section into core depth. Green line denotes the

position of the profile along which the grey scale curve (blue) was generated by averaging 30 pixels perpendicular to the line to reduce noise, and by producing ~2000 values along the line for this section. Red is floating average. The position where, according to the chosen settings, laminae are detected is marked by pink vertical lines. The software uses this position to calculate the thickness variability between the laminae and their total amount. In this example, the software detects 83 crossing, 42 maxima and 41 minima, implying that there are either 41 or 42 laminae present. The chosen settings for minimum and maximum count methods were 6 for full width half maximum (FWHM) Gaussian smoothing, 2 for minimum width and 0.04 for minimum height. For the zero crossing method, settings were 100 for FWHM, 4 for minimum width and 0.06 for minimum height. Further details are given in the text and in Weber et al.[6]

4. Fast ice inside Edisto inlet

Satellite analyses based on visible imagery indicate that the sea ice within Edisto Inlet is typically classified as persistent landfast ice, meaning it remains anchored to the coast rather than drifting with winds and currents like the pack ice outside the bay. This interpretation is supported by repeated in situ summer observations collected by Italian research expeditions inside the bay over the past 10 years. Variability in Edisto landfast ice is mainly characterized by seasonal retreat toward the inner coastline during melt events, usually followed by rapid recovery and subsequent advance toward the bay's mouth (Figure S4, February-March 2013). However, on occasion the open-water area released by the retreat of landfast ice may be partially infilled by drifting sea ice advected from outside the bay (Figure S4, 16th March 2024), which is typically displaced once the landfast ice resumes its seaward progression toward the bay mouth.

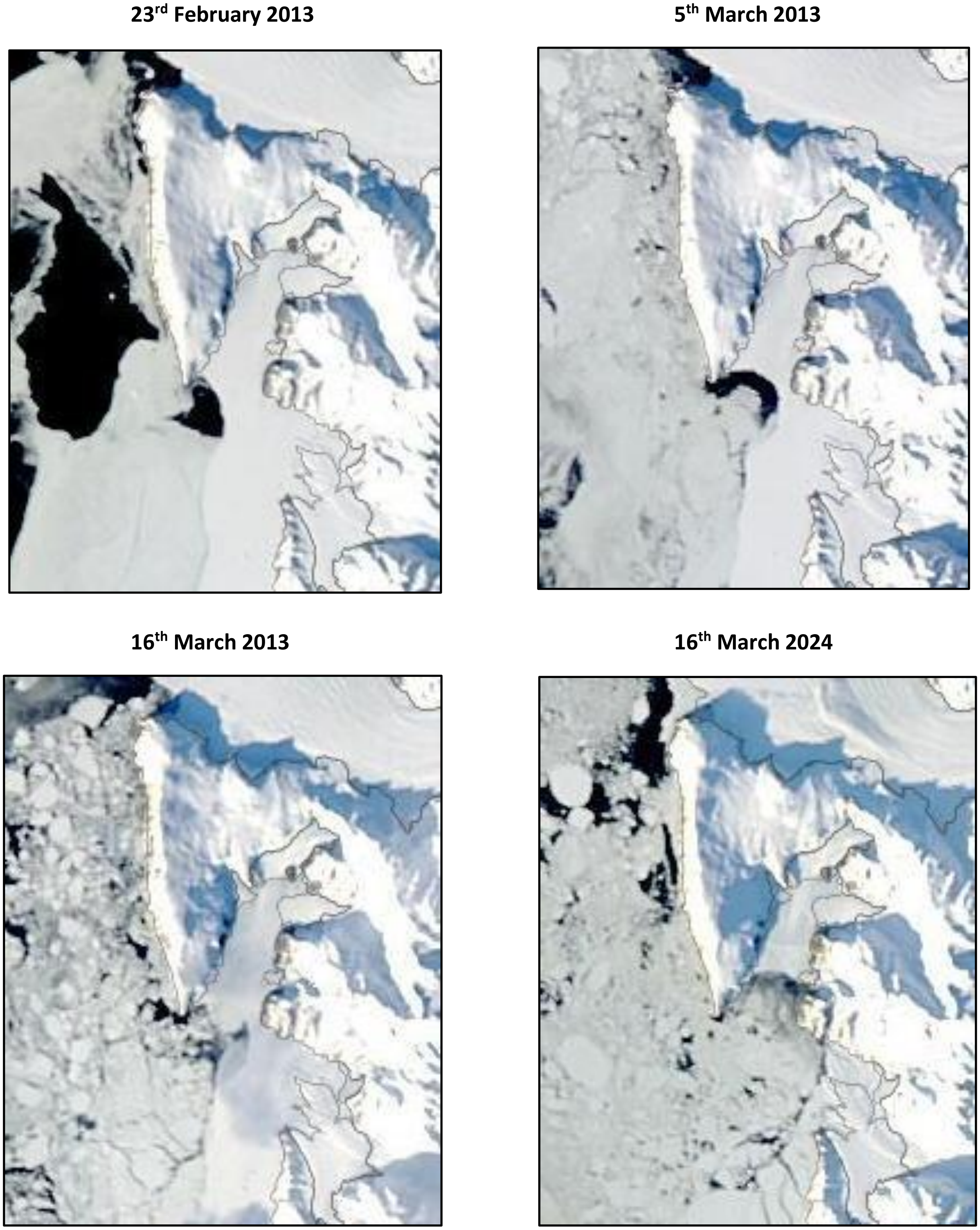

Figure S4. Satellite visible imagery illustrating the difference between pack ice and fast ice in Edisto inlet. The 2013 time series shows their evolution over time and the progressive loss of fast ice during

summer. Visible imagery analysis was conducted using MODIS Terra corrected-reflectance (true-color) products retrieved from the NASA Worldview platform (public domain; https://worldview.earthdata.nasa.gov).

4. Abrupt-solp4p experiment (Solp4p)

To understand the response of the Southern Hemisphere surface ocean and atmospheric circulation to a change in the solar constant, we analysed model outputs from the Cloud Feedback Model Intercomparison Project (CFMIP[7]), which is part of the sixth phase of the Coupled Model Intercomparison Project (CMIP6[8]). We used the CFMIP 'abrupt-solp4p' experiment (Solp4p), where fully coupled climate models initialised from pre-industrial (PI) conditions were perturbed with an abrupt 4% increase in the solar constant while keeping the distribution of solar energy across different wavelengths consistent with the PI experiment. These experiments were run for 150 years under this constant solar forcing, with other forcings, such as greenhouse gas concentrations, held at PI levels. Therefore, comparing the solp4p experiment with the PI control experiment allows us to examine changes in sea surface temperatures and sea ice cover around Antarctica brought about by increase in solar radiation.

We analysed the monthly mean atmosphere and ocean outputs from the Solp4p and PI experiments conducted using: the Community Earth System Model version 2 (CESM2) [9], the Institut Pierre-Simon Laplace Climate Model version for CMIP6 in the low-resolution configuration (IPSL-CM6A-LR) [10], the Canadian Earth System Model version 5 (CanESM5)[11], the Meteorological Research Institute Earth System Model version 2.0 (MRI-ESM2.0)[12] and the low-resolution configuration of Hadley Centre Global Environment Model version 3.1 (HadGEM3-GC31-LL) [13]. Table S2 specifies the horizontal resolutions of the atmosphere and ocean components of the models used in this study. For multi-model mean calculations, the atmosphere and ocean model outputs were re-gridded to commonly used 1°×1° resolutions using bilinear interpolation.

Model	Atmosphere resolution, km	Ocean resolution, km
CESM2	192×288	320×384
IPSL-CM6A-LR	144×143	362×332
CanESM5	64×128	290×361
MRI-ESM2.0	160×320	360×364
HadGEM3-GC31-LL	144×192	330×360

Table S2. List of the climate models used in this study and the horizontal resolutions of their atmosphere and ocean components.

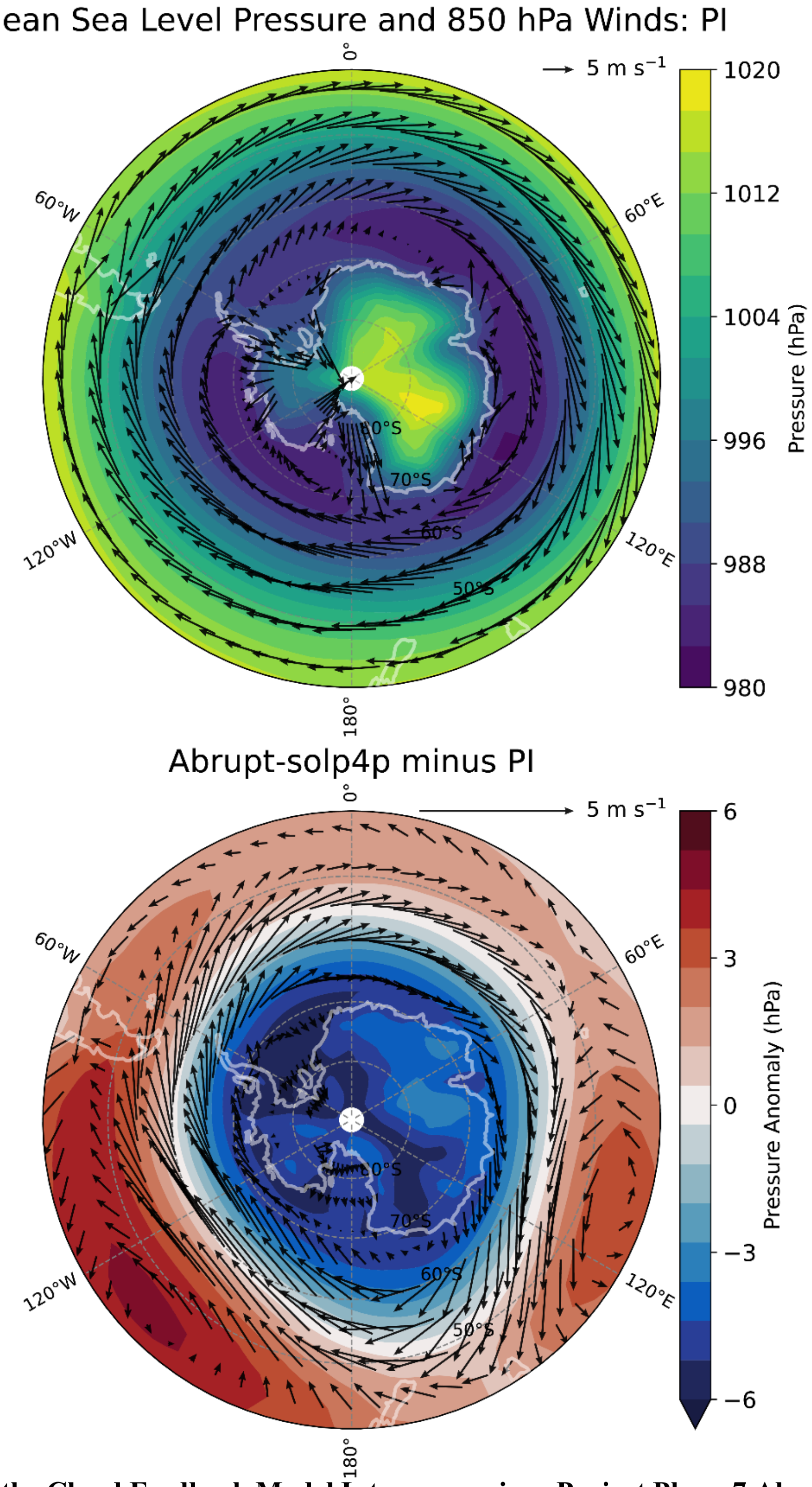

Figure S5. **Output from the Cloud Feedback Model Intercomparison Project Phase 7 Abrupt-solp4p (Solp4p) experiment, part of the Coupled Model Intercomparison Project Phase 6**. Multimodel mean sea level pressure (a) and wind vectors for pre-industrial (PI) and anomalies in the Abrupt-solp4p experiment (b).

References

1. Battaglia, F. *et al.* The discovery of the southernmost ultra-high-resolution Holocene paleoclimate sedimentary record in Antarctica. *Marine Geology* **467**, 107189 (2024).
2. Di Roberto, A. *et al.* First marine cryptotephra in Antarctica found in sediments of the western Ross Sea correlates with englacial tephras and climate records. *Sci Rep* **9**, 10628 (2019).
3. Heaton, T. J. *et al.* Marine20—The Marine Radiocarbon Age Calibration Curve (0–55,000 cal BP). *Radiocarbon* **62**, 779–820 (2020).
4. Hall, B. L., Henderson, G. M., Baroni, C. & Kellogg, T. B. Constant Holocene Southern-Ocean 14C reservoir ages and ice-shelf flow rates. *Earth and Planetary Science Letters* **296**, 115–123 (2010).
5. Tesi, T. *et al.* Resolving sea ice dynamics in the north-western Ross Sea during the last 2.6 ka: From seasonal to millennial timescales. *Quaternary Science Reviews* **237**, (2020).
6. Weber, M. E. *et al.* BMPix and PEAK tools: New methods for automated laminae recognition and counting—Application to glacial varves from Antarctic marine sediment. *Geochem Geophys Geosyst* **11**, 2009GC002611 (2010).
7. Webb, M. J. *et al.* The Cloud Feedback Model Intercomparison Project (CFMIP) contribution to CMIP6. *Geosci. Model Dev.* **10**, 359–384 (2017).
8. Eyring, V. *et al.* Overview of the Coupled Model Intercomparison Project Phase 6 (CMIP6) experimental design and organization. *Geosci. Model Dev.* **9**, 1937–1958 (2016).
9. Danabasoglu, G. *et al.* The Community Earth System Model Version 2 (CESM2). *J Adv Model Earth Syst* **12**, e2019MS001916 (2020).
10. Boucher, O. *et al.* Presentation and Evaluation of the IPSL-CM6A-LR Climate Model. *J Adv Model Earth Syst* **12**, e2019MS002010 (2020).
11. Swart, N. C. *et al.* The Canadian Earth System Model version 5 (CanESM5.0.3). *Geosci. Model Dev.* **12**, 4823–4873 (2019).

12. Yukimoto, S. *et al.* The Meteorological Research Institute Earth System Model Version 2.0, MRI-ESM2.0: Description and Basic Evaluation of the Physical Component. *Journal of the Meteorological Society of Japan* **97**, 931–965 (2019).

13. Roberts, M. J. *et al.* Description of the resolution hierarchy of the global coupled HadGEM3-GC3.1 model as used in CMIP6 HighResMIP experiments. *Geosci. Model Dev.* **12**, 4999–5028 (2019).
