# Supplementary material for: Late Holocene fast-ice dynamics around the Northern Victoria Land coast, Antarctica
Source: Nat Commun. 2026 Jan 20;17:604. doi: 10.1038/s41467-025-67781-7 (PMC12820077; doi:10.1038/s41467-025-67781-7)
Supplement: Supplementary file 2 — Description of Additional Supplementary Files [file 41467_2025_67781_MOESM2_ESM.pdf]

Dataset 1.

This dataset includes grey-scale measurements used to quantify sediment laminations in core TR17-08, derived following the methodology presented in the Methods. Line-scan images from each 1-m core section were downsampled from ~15,000 to ~2,000 pixels in length and converted to lossless BMP format for processing with the BMPIX tool A centre line was defined along each section, and grey-scale values were generated by averaging 30 pixels perpendicular to the line at every point, producing ~2,000 downcore measurements per section and a total of 29,001 grey-scale values for core TR17-08 (see Figure 3a).

Dataset 2.

This dataset includes automated lamination counts generated with the PEAK tool, part of the BMPIX/PEAK software package described in the Methods. These automated approaches provide objective, reproducible lamination counts and allow uncertainty assessment across multiple algorithms (see Figure 3b,c)

Dataset 3.

This dataset includes measurements of total organic carbon (TOC), stable carbon isotopes ($\delta^{13}C$), and the Antarctic sea-ice biomarker $IPSO_{25}$, together with diatom assemblage data. Sediment analyses were performed on both the dark and light intervals of the TR17-08 core (see Figure 4).

Dataset 4.

This dataset contains the results of a frequency analysis performed on grey-scale data to identify cycles and fluctuations in fast ice across multiple time scales (see Figure 7a). The analysis decomposes fast-ice variability into frequency-specific components, enabling the detection of both long-term trends (low-frequency signals) and shorter-term variations (high-frequency signals). For additional details on the frequency analysis, please refer to the *Methods* section.

Dataset 5.

This dataset provides near-daily observations of fast ice in Edisto Inlet and pack ice in the adjacent coastal region of the southwestern Ross Sea, Antarctica. The data are derived from thermal infrared measurements collected by NASA’s MODIS sensors on the Terra (since 2000)

and Aqua (since 2002) satellites. Level-1B MODIS granules (radiance, geolocation, and cloud-mask products) were processed to retrieve ice surface temperature (IST) and to distinguish reliably between sea ice and open water. Only scenes meeting strict cloud-mask confidence thresholds were retained, with additional visual screening for atmospheric contamination. The dataset spans austral summers from December 2000 to April 2024 and provides sea-ice classification for approximately 96% of days within Edisto Inlet and 79% in the surrounding coastal area (see Figure 8).
